# Validation of Accelerometry Data to Identify Movement Patterns During Agility Testing

**DOI:** 10.3389/fspor.2020.563809

**Published:** 2020-11-10

**Authors:** Wilshaw R. Stevens, Anthony M. Anderson, Kirsten Tulchin-Francis

**Affiliations:** Scottish Rite for Children, Dallas, TX, United States

**Keywords:** automation, timing, accelerometer, agility testing, stopwatch

## Abstract

**Purpose:** The purpose of this study was to develop an algorithm for the detection and timing of foot contact/off timing for each lateral repetition and assess the validity/reliability of the calculated timings.

**Methods:** Participants performed a modified Edgren Side Step Test in which they moved laterally along a 4-m path as quickly as possible while wearing an accelerometer on each ankle. Time of completion of each attempt was recorded using a stopwatch and digital video was obtained. Accelerometer-based (ACC) events were determined for the start of the test (START), foot contact at the end-line (FC) and the lifting of the foot when transitioning to the other direction (FO). Based on these ACC events the Overall, Split (ST) and Lag (LT) times were determined and compared to either the stopwatch or video-based timings (*p* < 0.05). The ACC event criterion was then applied by independent reviewers to assess inter/intra-rater reliability of identifying the events.

**Results:** There was no significant difference in ACC (12.37 ± 2.19 s) based Overall Time compared to the Stopwatch (12.42 ± 2.25 s, *p* = 0.34). Bland-Altman plots for ST and LT revealed very good agreement between the ACC time to the Video (ST: Bias = 0.11 s, LOA −0.57 to 0.79; LT: Bias = −0.11 s, LOA −0.43 to 0.22). Intra and inter-rater reliability was moderate to excellent for all reviewer identified events.

**Conclusions:** This study demonstrates methodology to identify ACC based timings during an agility test. The inclusion of an accelerometer supplements standard timing options with the added benefit of assessing sided split and lag times.

## Introduction

Studies have been conducted demonstrating the accuracy and reliability of collecting overall performance time (including split times) in various functional agility tests (Lundquist, 2007; Hetzler et al., 2008; Mayhew et al., 2010). In general, stopwatch times collected among individual raters have been shown to be highly reliable with differences reported between 0.04 and 0.41 s (Lundquist, 2007; Hetzler et al., 2008; Mayhew et al., 2010). While stopwatches have been shown to be a cost effective means of measuring performance time, advancements in wearable technology, such as accelerometers and inertial measurement units, allow for greater detail in timing and performance analysis (Li et al., 2016; Camomilla et al., 2018).

Low cost, low power wearable sensors can further identify strategies associated with optimal performance during functional testing by providing quantitative measurements of movement including spatiotemporal parameters, segment accelerations, estimates of mechanical loading and classification of the quality of movement (Godfrey et al., 2008; Kavanagh and Menz, 2008; Rowlands and Stiles, 2012; Barnes et al., 2017; Zaferiou et al., 2017). For example, Zaferiou et al. (2017) demonstrated the utility of placing sensors on both feet during an agility drill to objectively quantify foot contacts with the ground and further identify strategies associated with higher performance. Signal analysis of these high performers revealed that they performed the test using strategies involving sharper turns and larger changes in body speed (Zaferiou et al., 2017). The utility of this methodological approach to quantify mechanical movement patterns during a fitness test has been demonstrated in children (Barnes et al., 2017). High performing children demonstrated unique movement patterns which included linearly increasing speed (Barnes et al., 2017).

The most common methods to record completion times during functional performance evaluations use a stopwatch or timing light. Depending on setup, these data collection tools may also allow measurements of split times during each lap/repetition of the task, in addition to overall completion times. As wearable sensors become more common place during functional testing, the development of automated algorithms to aid in data processing/analysis improves the time efficiency from data collection to dissemination of performance results. To be successful, these tools must be validated, have high reliability and be easily administered with minimal training. Toward this effort, the current study aimed on developing a method to assess foot contact/off using ankle-worn accelerometers during a lateral agility test. The purpose of this study was therefore to: (1) develop an algorithm for the detection and timing of foot contact/off timing for each lateral repetition, and (2) assess the validity and reliability of the calculated timings using the new method.

## Materials and Methods

Participants in this study are a subset of a larger cohort enrolled as part of an institutional review board approved study assessing balance and agility in healthy individuals over the age of 5 years. Participants were excluded from testing if they had underlying medical/health conditions such as neurological or orthopedic conditions, asthma, current joint pain, and/or lower extremity or back injury/pain, within the last 6 months (required a doctor's visit), or surgery within 1 year of testing. All eligible participants provided informed consent, and assent was obtained from participants over the age of 10 years.

### Modified Edgren Side Step Test

The lateral agility test performed in this study was the Edgren Side Step Test (ESST) which was introduced in 1932 as an objective test to assess lateral mobility, and has been shown to be a useful functional ability evaluation tool in both athletes and non-athletes (Edgren, 1932; Semenick, 1994; Harman and Pandorf, 2000; Chaouachi et al., 2009; Gailey et al., 2013; Raya et al., 2013). Assessment tools such as the ESST have been shown to be reliable, and detailed protocols for administering this test are readily available to clinicians and researchers (Semenick, 1994; Harman and Pandorf, 2000; Chaouachi et al., 2009). The original test is administered over a 10 s period and performance is graded based on how many cones/lines are crossed.

Participants performed a modified version, the mESST, adapted to provide time-based assessments of the demand on each leg, rather than a singular distance-based evaluation. Participants were instructed to start in a standing position with both feet outside one end-line. At the “go” command, participants side stepped laterally (going to their right first) across 4-m toward the opposite end-line, quickly stopped and side stepped laterally in the opposite direction. This “down-and-back” was counted as one repetition and this was performed three times. Only one foot was required to touch or cross each end-line. The time to complete the course was determined using a stopwatch. Each participant was given two attempts at the mESST. A trial was disqualified if participants performed any of the following: (1) failed to touch/cross the end-line, (2) crossed their legs while side-stepping, or (3) failed to keep their pelvis pointing forward.

### Accelerometer Initialization and Setup

Participants were fitted with Actigraph GT3X+ (Actigraph, Pensacola, FL, USA) accelerometers on each ankle, set to record three dimensional acceleration (g) at 100 Hz. Both Actigraphs were programmed in the Actilife software (Actigraph, Pensacola, FL, USA) simultaneously using the group initialization option, which ensured that they were on the same time clock. Once the Actigraphs were successfully initialized, they continuously recorded time-stamped acceleration data until the devices were plugged into the computer and downloaded. At this point, data recording to the internal memory stopped. The devices were worn on the lateral border of the ankle, directly above the malleolus of both legs. The Actigraphs were orientated such that the vertical axis was in the superior/inferior plane, the horizontal axis was in the medial/lateral plane, and the perpendicular axis was in the anterior/posterior plane of the participant.

### Testing Procedures and Data Processing

Two research team members were involved in each participant test. The first team member gave each participant instructions, demonstrated the mESST and recorded the participant's overall completion time using a standard stopwatch capable of recording to the 1/100^th^ of a second. The second researcher recorded the time of day of the start and end of each mESST attempt, so that the acceleration data of that attempt could be extracted for data processing purposes. The start time was recorded on the “go” command given by the first team member and the end time was recorded when the subject crossed the final end-line. Digital video was recorded using a Sony Camcorder collecting high definition video data, at a standard sample rate of 30 frames per second. The video camera was positioned in the middle of the testing area at a distance of ~20 feet in front of the participant, to ensure that end-lines could be visualized. The video recording was started just prior to the first team member giving the “go” command to the participant and was stopped well after the participant had crossed the final end-line. This was usually after the second team member had documented the end time of day. This video would be used later to verify timing events of the test (start, end, and transitions).

**Figure 1:**
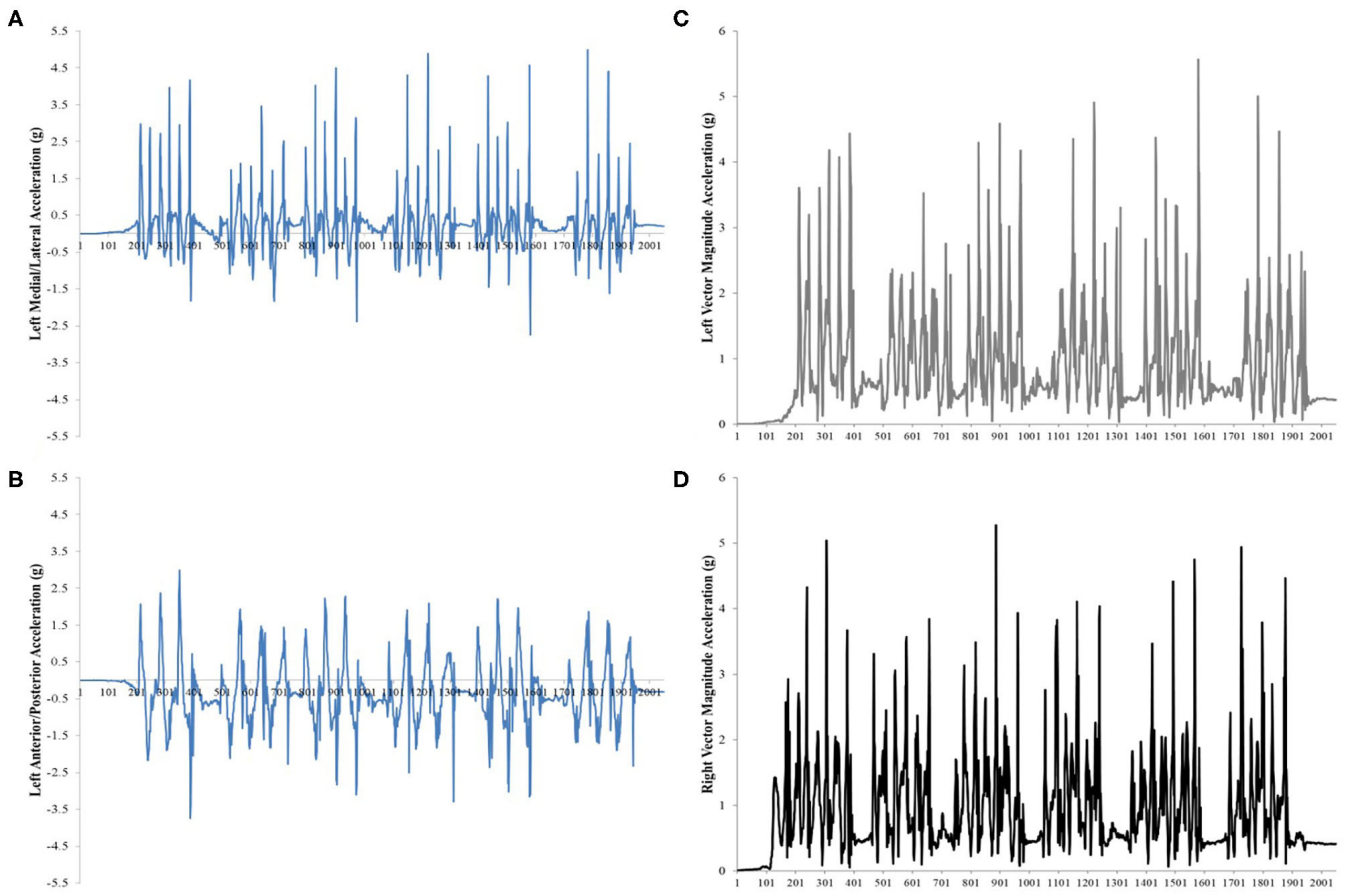
**(A–D)** Example acceleration data (g) of one test attempt of the modified Edgren Side Step Test (mESST). **(A)** Left Medial/Lateral acceleration, **(B)** Left Anterior/Posterior acceleration, **(C)** Vector magnitude of the Left Medial/Lateral and Anterior/Posterior acceleration data (g), **(D)** Vector magnitude of the Right Medial/Lateral and Anterior/Posterior acceleration data (g).

Upon completion of the test, the acceleration data were downloaded and exported to a Microsoft Excel file using the Actilife software. A custom written MATLAB code (Mathworks, Natick, MA, USA) was written to separately extract each attempt of the test, according to the time of day that was recorded at the start and end of each attempt. To ensure that the entire attempt was analyzed, acceleration data from two seconds prior to the recorded “start” time of day and three seconds after the recorded “end” time of day was extracted. The left and right sided data were saved separately (Figures 1A,B). To account for accelerometer placement, a gravitational offset was subtracted from both the medial/lateral and anterior/posterior planes individually. This offset was calculated by taking the average of the acceleration of each plane during two seconds of quiet standing prior to the start of the test attempt. These offsets were calculated for the right and left sides separately.

A two-dimensional vector magnitude (VMag) of the acceleration (g) at each data point in the medial/lateral and an anterior/posterior plane was then calculated for both sides. This bi-planar vector was used as it was observed that participants had an external foot progression angle while side stepping. This resulted in accelerations occurring not only in the medial/lateral plane (which was expected during a lateral side-step) but also in the anterior/posterior plane (Figure 1B). The VMag of each attempt was plotted so that each individual data point from the start to the end of that attempt could be easily identified (Figures 1C,D).

### Development of Event Criterion

**Figure 2:**
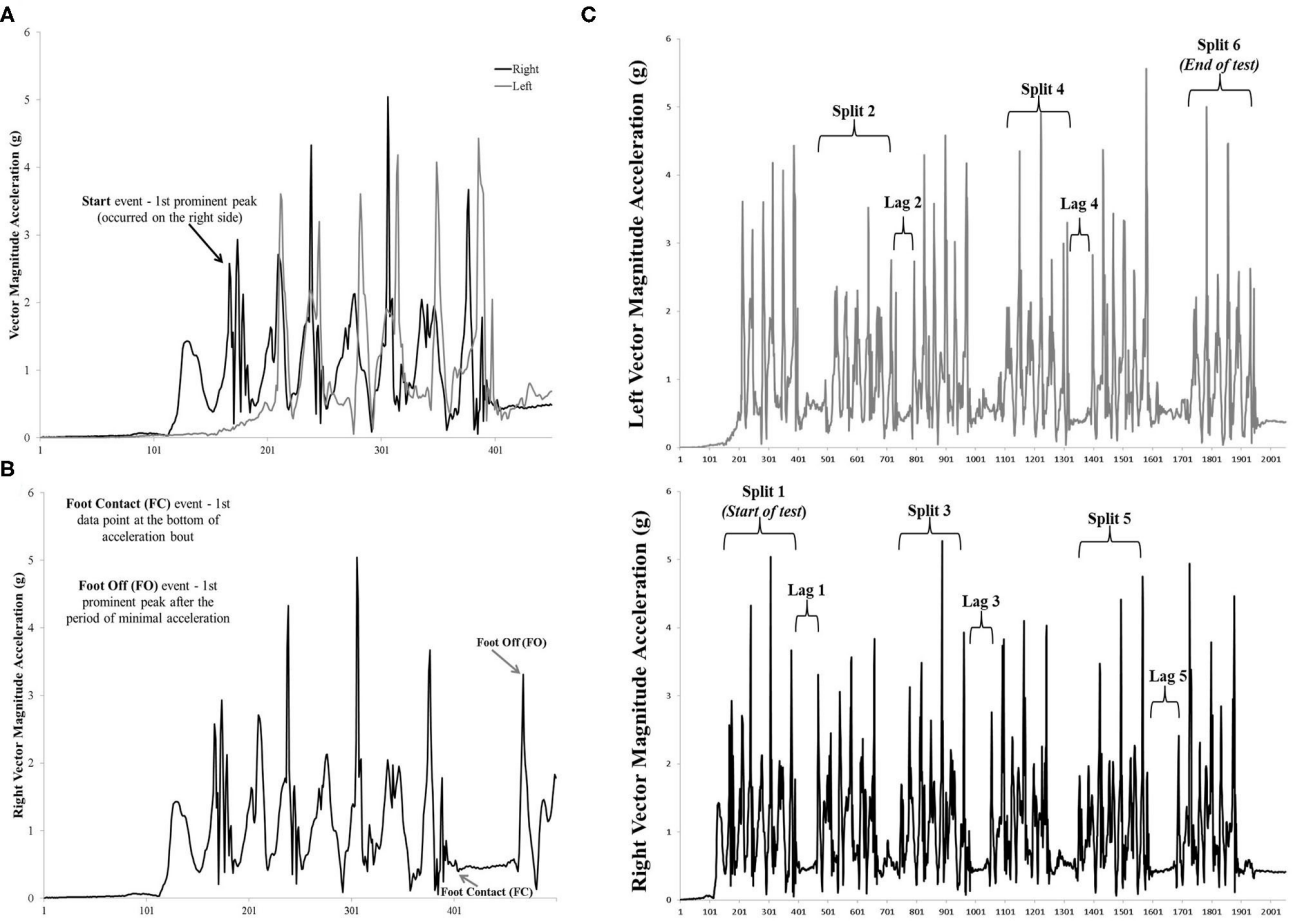
**(A–C)**. Start, Foot Contact (FC) and Foot Off (FO) events identified on the vector magnitude of the acceleration data. **(A)** Start identified as the first prominent peak of the acceleration data which occurred on the right side in this example. **(B)** FC event identified as the first data point at the bottom of acceleration bout on the right side acceleration data. FO event identified as the first prominent peak after the period of minimal acceleration on the right side acceleration data. **(C)** Split time and Lag time periods identified on the vector magnitude of acceleration data from the right and leg left during one attempt of the modified Edgren Side Step Test (mESST). Split time and Lag time duration periods were calculated from the Foot Contact and Foot Off events identified in the acceleration signal of either the right or left leg. For one repetition of the mESST there are six Split Times (subject travels from one end-line to the opposite end-line) and five Lag times (time subject took to transition to movement in the opposite direction).

Using a group of 11 participants (age range 7–23 years old, six females, height range 1.2–1.7 m, weight range 22–71 kg), one attempt of their mESST was reviewed by two raters who identified foot contact and foot off events developed from a visual analysis of the periodicity of the VMag acceleration signal and a frame by frame analysis of the video data. During the initial event criterion development, the raters worked together to identify the foot events and establish a consensus of the event criterion. Although each participant began the test moving toward their right, it was observed that participants' reactions to the “go” command varied slightly. The start of the test (START) was therefore defined as the earliest instance of an acceleration burst defined by the VMag data point of the first prominent peak, regardless of side (Figure 2A).

For the first pass, as the participant traveled to the opposite end-line, the right foot was considered the lead foot, and there was a distinct grouping of acceleration (acceleration bout). As the right foot landed across the opposite end-line (foot contact, FC), the first data point at the bottom of the acceleration bout (VMag approximately equal to 0 g) was identified as a FC (Figure 2B). Immediately following this bout was a period of minimal acceleration (VMag plateau around 0 g) in which the right foot was on the ground and the body is transitioning to side step toward the opposite end-line. Foot off (FO) was identified as the data point of the first prominent peak after the period of minimal acceleration (Figure 2B). The left foot is now considered the lead foot and the same event definitions were applied to identify a FC event followed by a FO at the opposite end-line. This process of alternating between the right/left feet was repeated for each pass with the sixth pass (third left foot leading pass) detecting the end of the test. The end of the test (END) was identified using the VMag acceleration signal of the left foot using the FC criteria, which was the first data point at the bottom of the acceleration bout (VMag approximately equal to 0 g).

A total of 12 events were identified across the entire test repetition. On the right, there were six events, comprised of three FC and three FO, while on the left there were five events which comprised of three FC (one of which was the END event) and two FO. As previously stated, the START event could be detected on the either the right or left.

### Time Measure Definitions

Accelerometry data time measures were calculated by subtracting event data point numbers and dividing the result by 100 (accelerometer sample rate). For example, the Split Time (ST) of the first pass (start of test to opposite end line), was calculated by subtracting the START event from the FC event of the right foot (leading leg) and dividing that result by 100 [(START-FC)/100] (Figure 2C). All subsequent ST were defined as the travel time between end-lines and were calculated by subtracting the FO event (beginning of a new pass) from the termination of that pass, identified as the FC of the leading foot [(FO-FC)/100]. Lag Time (LT) was defined as the transition time as the participant's foot was on the ground and they transitioned movement to the opposite direction. LT was measured by subtracting the FC of the leading leg from the FO of that same leg [(FC-FO)/100]. In an effort to automate the time calculations, a Microsoft Excel document was created to automatically calculate the Overall Time, ST, and LT based on the accelerometer event data points that were selected.

In addition to the accelerometer-based time measures, the digital video was reviewed by the two raters working together and similar times were determined for each pass. In summary, for each test attempt, the overall time was determined using three different methods for comparison—stopwatch, digital video, and accelerometer data. Six STs and five LTs were determined by both the digital video and the accelerometers.

### Event Criterion Validation

For the 11 attempts that were used to develop the event criterion, the accelerometer based Overall times were compared to the stopwatch and video based times using a repeated measures analysis of variance (ANOVA with three levels) and a *post-hoc* paired *t*-test for each pair of comparison means (alpha = 0.05). The level of agreement between the accelerometer to the stopwatch and video based times was assessed using the Bland-Altman method. Bland-Altman analyses were conducted to assess the bias (mean differences) between the accelerometer (ACC) and video (Video) time-based measures. The 95% limits of agreement (LOA) were assessed, and the Bland-Altman plot allowed for further investigations of the bias in relation to the duration of time (Overall, ST and LT times).

Spearman rank correlation coefficients between the ACC and both the Stopwatch and Video times were also determined as the data was not normally distributed. Intraclass correlation coefficient [ICC (2, 1), 95% Confidence Interval, CI] was determined among the Stopwatch, ACC and Video for the Overall time comparison. Correlations were classified according to the guidelines suggested by Portney and Watkins (2008).

### Testing of Event Criterion by Independent Reviewers

Four independent reviewers were trained by the two initial raters who developed the event criterion. Reviewers were given an ~10 min training session where the event criterion were explained and two example datasets were used to place each event with group consensus. A third example dataset was used for reviewers to independently apply the criterion and each reviewer received feedback from the raters on the event selections. Following the completion of this training session, each reviewer was provided a reference sheet with the event criterion definitions to use for further assessments.

For evaluation of the event criterion, all reviewers (*n* = 6) independently identified the accelerometer events using the guidelines outlined above, on seven additional attempts of the mESST (age range 7–19 years old) not previously used in the event criterion development process. To assess intra-rater reliability, 94 data points across seven attempts were identified by each reviewer on two separate occasions, three to seven days apart. Inter and intra-rater reliability using intraclass correlation coefficient [ICC (2, 1), 95% Confidence Interval, CI] was determined for all acceleration-based data points. All statistical analyses were run using SPSS (version 24, IBM Inc., Chicago, IL, USA) with statistical significance was set at alpha of 0.05, as applicable.

## Results

### Comparison of Methodologies for Overall Time, Split Time, and Lag Time

The Overall, Split (ST) and Lag (LT) times for the 11 attempts of the test used to develop the event criterion were compared across methodologies. The results of the repeated measures ANOVA on the Overall times showed that there was a significant difference across methodologies (*p* < 0.01). *Post hoc-*paired t-tests were run and revealed that there was no significant difference in the average Overall Time based on the ACC measures (12.37 ± 2.19 s) compared to the Stopwatch (12.42 ± 2.25 s, *p* = 0.34). The average Overall time derived from the Video (12.20 ± 2.15 s) was significantly shorter than the ACC (12.37 ± 2.19 s, *p* = 0.02) and Stopwatch times (12.42 ± 2.25 s, *p* < 0.01). Bland-Altman plots revealed very good agreement between the ACC time-based measure to the Stopwatch (Bias = −0.05 s, LOA −0.36 to 0.26) and Video (Bias = 0.17 s, LOA −0.22 to 0.56) Overall times (Supplementary Figures 1a,b).

ST and LT were compared using Bland-Altman plots between the ACC and Video methodologies. Bland-Altman plots for ST and LT revealed very good agreement between the ACC time to the Video (ST: Bias = 0.11 s, LOA −0.57 to 0.79; LT: Bias = −0.11 s, LOA −0.43 to 0.22) (Supplementary Figures 2a,b).

There was excellent interrater reliability in the Overall Time as measured by the Stopwatch, ACC and Video [ICC (2, 1) = 0.994, (CI 0.975, 0.998)]. There was a very strong correlation between the ACC measured Overall time and Stopwatch (ρ > 0.99, *p* < 0.01) and Video based time measures (ρ > 0.99, *p* < 0.01) (Supplementary Figures 3a,b). The ST time for ACC was moderately correlated to the Video (ρ = 0.60, *p* < 0.01) with fair correlation between the two methodologies for LT (ρ = 0.39, *p* < 0.01) (Supplementary Figures 4a,b).

### Inter/Intra-rater Reliability of Identifying Accelerometer Data Points

Six reviewers identified the accelerometer data point numbers (START, Foot Contact (FC) and Foot Off (FO) and END data points) on seven attempts of the mESST. Intra-rater reliability was good to excellent for the START (ICC range 0.70–1.00) and END data points (ICC range 0.99–1.00) (Table 1). For all the data points identified, intra-rater reliability was moderate to excellent across the raters (ICC range 0.70–1.00). For all the FC and FO data points (represented by the calculated ST and LT), intra-rater reliability was moderate to very good (ICC range 0.68–0.98) (Table 1).

**Table 1:** Intra-rater ICC^*^ with 95% Confidence Interval (CI) of accelerometer defined times derived from events manually identified on acceleration data during seven separate test repetitions of the mESST; Data Point (DP).

**Reviewer**	**Start DP**	**End DP**	**Across 12 (DP)^**∧**^**	**Split time**	**Lag time**
1	0.70 (−0.05, 0.94)	1.00 (0.98, 1.00)	0.70 – 0.99	0.90 (0.81, 94)	0.84 (0.70, 0.91)
2	0.98 (0.92, 1.00)	1.00 (1.00, 1.00)	0.99 – 1.00	0.98 (0.97, 0.99)	0.94 (0.89, 0.97)
3	0.99 (0.93, 1.00)	1.00 (1.00, 1.00)	0.99 – 1.00	0.98 (0.97, 0.99)	0.97 (0.94, 0.98)
4	1.00 (1.00, 1.00)	1.00 (1.00, 1.00)	1.00 – 1.00	0.90 (0.82, 0.94)	0.68 (0.45, 0.82)
5	0.99 (0.96, 1.00)	0.99 (0.97, 1.00)	0.99 – 1.00	0.94 (0.90, 0.97)	0.95 (0.91, 0.98)
6	0.99 (0.97, 1.00)	1.00 (1.00, 1.00)	0.99 – 1.00	0.94 (0.85, 0.97)	0.82 (0.47, 0.93)

* Two-way mixed model with absolute agreement ICC (2, 1).

∧ *Range of ICC values across the 12 data points*.

There was good interrater reliability in identifying the START (ICC = 0.85, CI 0.6, 0.97) and END data points (ICC = 0.99, CI 0.98, 1.00) (Table 2). For all the data points identified, inter-rater reliability was good to excellent across the raters (ICC range 0.85–0.99). For all other FC and FO data points (represented by the calculated ST and LT), interrater reliability was good [ST: ICC 0.89 (CI 0.82, 0.93); LT: ICC 0.78 (CI 0.64, 0.87)] (Table 2).

**Table 2:** Inter-rater ICC^*^ with 95% Confidence Interval (CI) of accelerometer defined times derived from events manually identified on acceleration data during seven separate repetitions of the mESST; Data Point (DP).

**Start DP**	**End DP**	**Across 12 DP^**∧**^**	**Split times**	**Lag times**
0.85 (0.66, 0.97)	0.99 (0.98, 1.00)	0.85–0.99^∧^	0.89 (0.82, 0.93)	0.78 (0.64, 0.87)

* Two-way mixed model with absolute agreement ICC (2, 1), six raters.

∧ *Range of ICC values across the 12 data points*.

## Discussion

This study developed accelerometer based criterion to identify the foot contact/off timings during the mESST, in an effort to improve upon stopwatch based measures for a more detailed assessment. These time measures included the Overall, Split and Lag times throughout the test. The ACC derived time measures had a high level of agreement to the stopwatch and video based times and allow for a more robust analysis of movement patterns based on the accelerometer signal. The criterion developed was implemented by independent reviewers on a separate dataset and overall inter/intra-rater reliability was very good.

In the development of the ACC event criterion, the Overall Times varied from 9.40 to 16.38 s (*n* = 11 participants). It was important that these times covered a wide range in order to determine if the unique ACC signal characteristics would be identifiable across various speeds and different functional performance levels. Additionally, the mESST can be assessed in individuals across a wide range of ages. Our lab is focused on assessing short- and long-term functional outcomes in the pediatric population, therefore, the age range of the participants chosen for the criterion development covered our target population.

A stopwatch is most commonly used for measuring time during an agility test, so as a benchmark it was important that the Overall Time calculated from the ACC events was comparable to the stopwatch recorded time. The Bland-Altman plot comparing the ACC derived Overall time to the stopwatch showed that the ACC time was ~0.05 s sooner. Mayhew et al. (2010) reported a maximum hand time difference of 0.19 s between timers using stopwatches. The ACC derived Overall Time of the present study was well within that range and has reasonable agreement when compared to a stopwatch. The ACC derived Overall Time was on average 0.17 s sooner than the video based time and this was significantly different. Nevertheless, this difference also fell under what has been previously reported. ACC derived Overall Time was based on the identification of the START and END data points on the acceleration data and trained reviewers had very good reliability (ICC > 0.80, Table 2) identifying these points. In five of the six reviewers, test re-test reliability was >0.90.

The discrepancy between the ACC and the video based timings were likely due to the slower video frame rate (30 video frames per second vs. 100 acceleration data points per second), which is considered a limitation of the current study. A high speed video camera would allow for more frames of movement to be analyzed which may result in improved agreement between the video and ACC timings. Other timing alternatives include electronic timers (timing lights, timing switches, etc.) which have a higher cost and increased setup time and have been previously compared to hand-held stopwatch time (Hetzler et al., 2008; Mayhew et al., 2010), however these were not used in the current study. Previous research has shown though that hand-held timers are a reliable option when the electronic option is not available (Lundquist, 2007; Hetzler et al., 2008; Mayhew et al., 2010).

The inclusion of accelerometers on both legs while a subject performs the mESST provides a unique opportunity to quantify ST, the time taken to travel from one end-line to the opposite end-line, along with LT, measured as the time needed for a participant to transition at the end-line and change directions. With each participant completing three “down and back” passes between end-lines there was the opportunity to identify six STs and five LTs which were categorized in either the right or left leg acceleration data, depending on the direction of movement. These additional timings allow for quantification of asymmetry in performance between the legs, as with each change of direction, the origination of propulsion to move laterally also changes. Therefore, the foot contact and foot off events at the end of each pass were of great importance. The ACC derived ST and LT were compared to timings based on video data collected using a traditional low-cost camcorder with standard features, rather than a specialized high frame rate video camera. The ACC derived ST were significantly longer than the video based timings and the Bland-Altman plots showed that the mean difference was 0.11 s. Previous studies have shown that among timers the absolute errors in ST ranged from 0.12 to 0.16 s in various agility tests (Lundquist, 2007; Hetzler et al., 2008). In the present study, the ACC derived ST difference to the video based timings was below that has been previously reported. Therefore, the authors were satisfied that the identified points were valid for categorization of ST in the mESST. Good inter-rater (ICC ranged from 0.85 to 0.99) and intra-rater (ICC ranged from 0.70 to 1.00) reliability was observed among reviewers in identifying the acceleration data points associated with Foot Contacts and Foot Offs, which were used to calculate the ST.

Due to the periodicity of the acceleration data during the lateral movements of the mESST, a distinct plateauing of the acceleration signal was seen as the foot contacted the ground at the end-line in preparation to change direction. This LT period was a unique identifiable component of integrating accelerometers with the mESST. The ACC LT were significantly shorter (Bias = −0.11 s, Supplementary Figure 2b) than the video LT and a poor correlation (ρ = 0.39, *p* < 0.01) was seen between the methods. Lag times are extremely brief; for example in the mESST attempts used to derive the ACC LT criterion, the average LT was 0.36 s. The identification of events of this short period of time requires both precision and accuracy. While the reliability of the video was not evaluated within the scope of this study, all video measures were identified as a consensus between two raters. However, the identification of events was difficult on the video, as with each frame, there was subtle movements of the foot that were lost due to the slow video frame rate (30 Hz) compared to the ACC data (100 Hz). The ACC data clearly showed the diminished movement as the foot stayed on the ground during the transition period to change direction which was not as easily identifiable on the video data. Despite the advantages of the accelerometer data, interrater reliability of lag times, while still considered good, was the lowest at 0.78 with intra-rater ICCs ranging from 0.68 to 0.97 among the reviewers. Similar to the ST assessment, five of the six reviewers had intra-rater reliability above ICC > 0.80.

Based on these results the assessment of accelerometry data with the mESST is a valid approach to identify multiple timings (Overall Time, Split Times and Lag Times) throughout the test. The proposed accelerometer defined points were developed to supplement the stopwatch methodology that is commonly used when administering this test. Accelerometer define criterion required minimal training (<10 min) and results showed very good validity, reliability and repeatability. With advancements in wearable technology it is becoming more common to include accelerometers as a means of quantifying subject's movement patterns during clinical tests with greater detail. The inclusion of wearable sensors has been used to identify specific movement patterns consistent within high performers compared to poor performers during various agility courses (Li et al., 2016; Barnes et al., 2017; Zaferiou et al., 2017; Camomilla et al., 2018).

Future work should include the application of the current methodology to assess individuals with various functional impairments. The use of individually sided split and lag times may provide clinically useful evaluations in those with unilateral musculoskeletal issues for example, however these measures require additional work to determine the minimal detectable change. Nevertheless, implementation of the accelerometer does not require a significant amount of additional testing time and a Microsoft Excel template was used in this study to automatically calculate the timings as the accelerometer define points were inputted. The proposed criterion for the identification of accelerometer defined points during the mESST is a reliable option for the quantification of event timings. Researchers analyzing the mESST may wish to supplement standard timing options of overall completion time with sided split and lag times determined using the developed accelerometer defined criterion and proposed methodology.

## Data Availability Statement

The raw data supporting the conclusions of this article will be made available by the authors, without undue reservation.

## Ethics Statement

The studies involving human participants were reviewed and approved by University of Texas Southwestern Medical Center. Written informed consent to participate in this study was provided by the participants' legal guardian/next of kin.

## Author Contributions

WS, AA, and KT-F: substantial contributions to the conception or design of the work, or the acquisition, analysis, or interpretation of data for the work, drafting the work or revising it critically for important intellectual content, and final approval of the version to be published. All authors contributed to the article and approved the submitted version.

## Conflict of Interest

The authors declare that the research was conducted in the absence of any commercial or financial relationships that could be construed as a potential conflict of interest.
