# Supplementary material for: Validation of Accelerometry Data to Identify Movement Patterns During Agility Testing
Source: Front Sports Act Living. 2020 Nov 10;2:563809. doi: 10.3389/fspor.2020.563809 (PMC7739769; doi:10.3389/fspor.2020.563809)
Supplement: Supplementary file 2 [file Image_2.pdf]

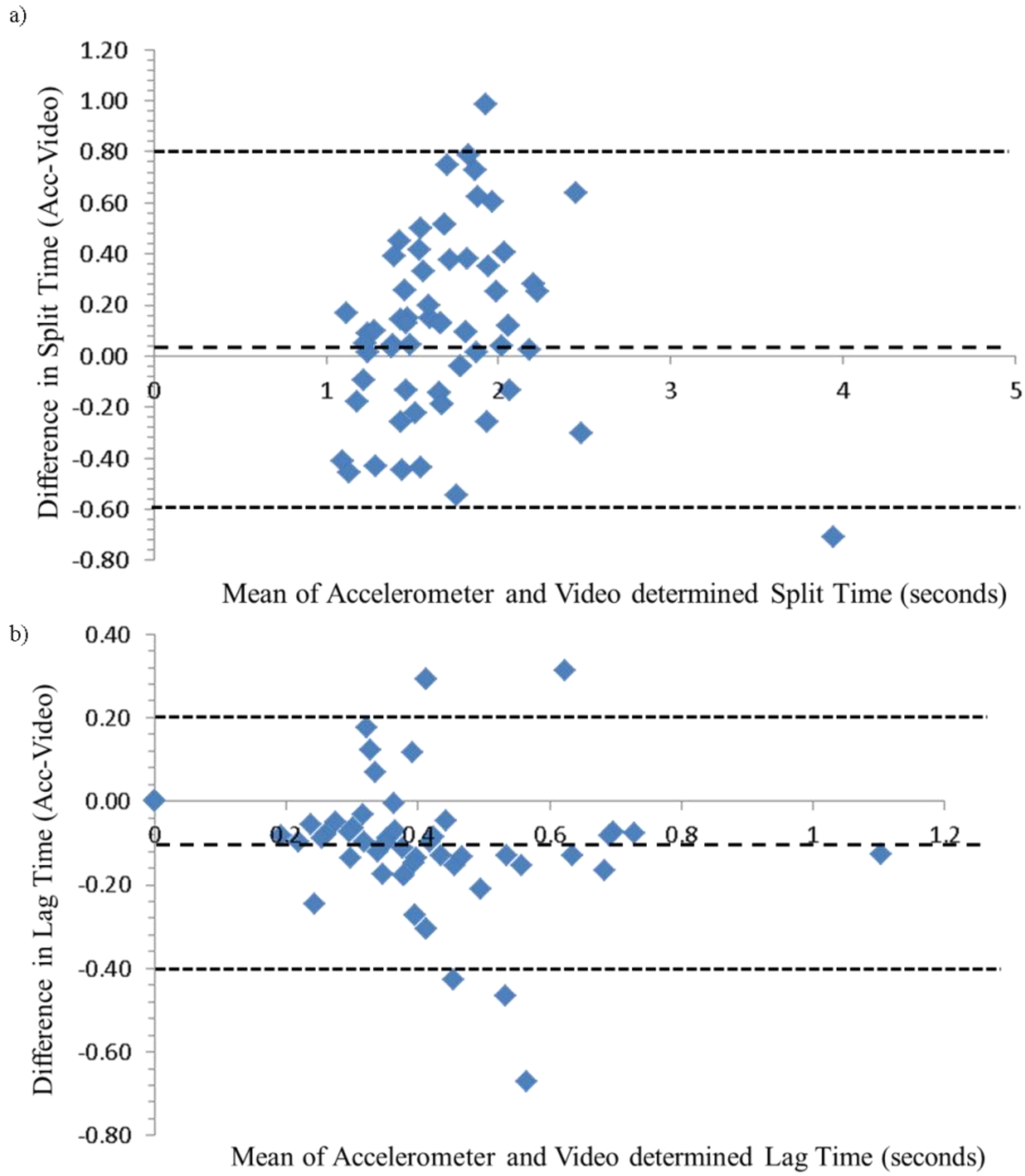

Supplementary Figure 2a, 2b. Bland-Altman plot depicting the agreement between Accelerometer (Acc) and Video derived Split Time and Lag Time (seconds). The dashed line represents the mean difference between the measures (Acc – Video derived times), and dotted lines represent the upper and lower limits of agreement.
