# Supplementary material for: Validation of Accelerometry Data to Identify Movement Patterns During Agility Testing
Source: Front Sports Act Living. 2020 Nov 10;2:563809. doi: 10.3389/fspor.2020.563809 (PMC7739769; doi:10.3389/fspor.2020.563809)
Supplement: Supplementary file 3 [file Image_3.pdf]

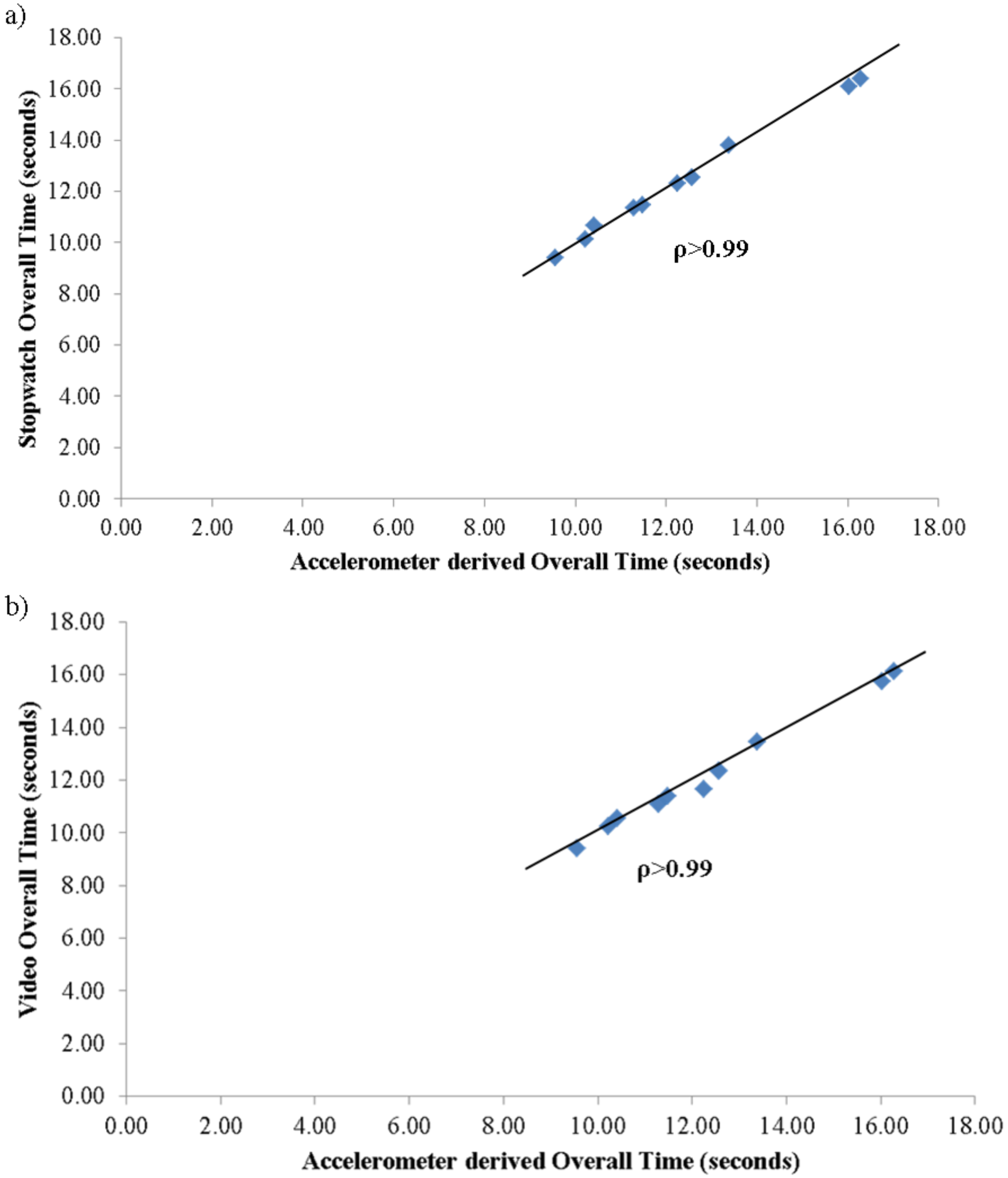

Supplementary Figure 3a, 3b. Spearman rho correlations for Overall Time (seconds) measured using a Stopwatch, Video and the Accelerometer derived event criterion. Excellent correlations ($\rho>0.90$, $p<0.01$) were observed when comparing the Accelerometer derived Overall Time to the Stopwatch and Video times.
