# Supplementary material for: Validation of Accelerometry Data to Identify Movement Patterns During Agility Testing
Source: Front Sports Act Living. 2020 Nov 10;2:563809. doi: 10.3389/fspor.2020.563809 (PMC7739769; doi:10.3389/fspor.2020.563809)
Supplement: Supplementary file 4 [file Image_4.pdf]

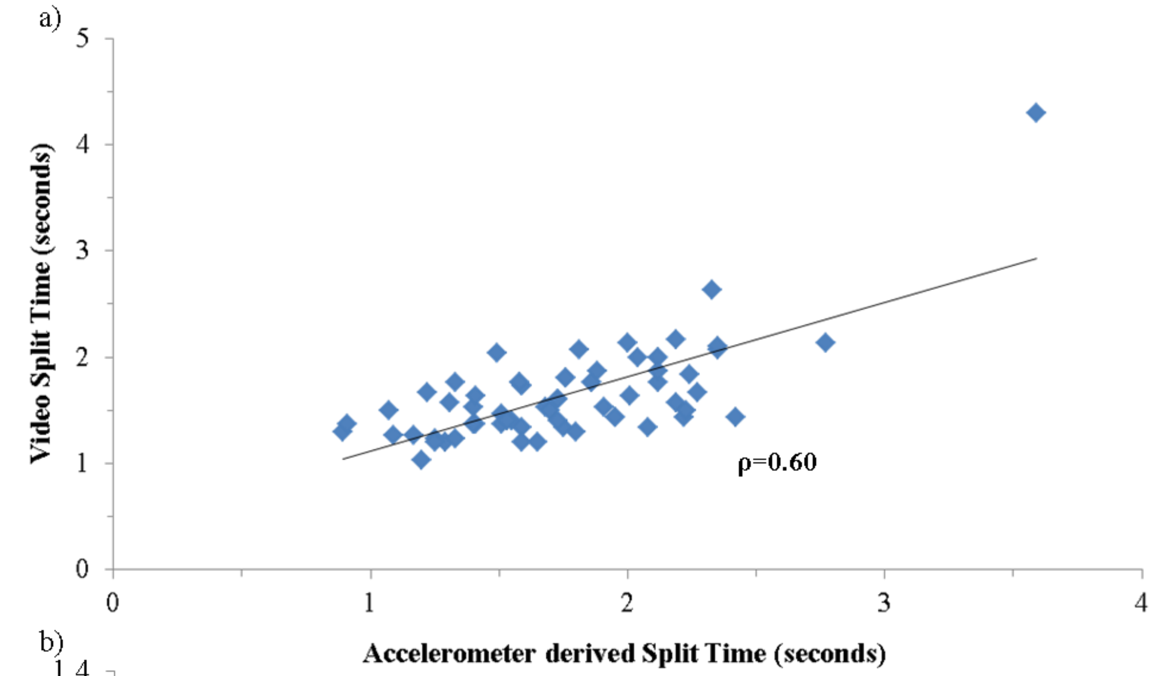

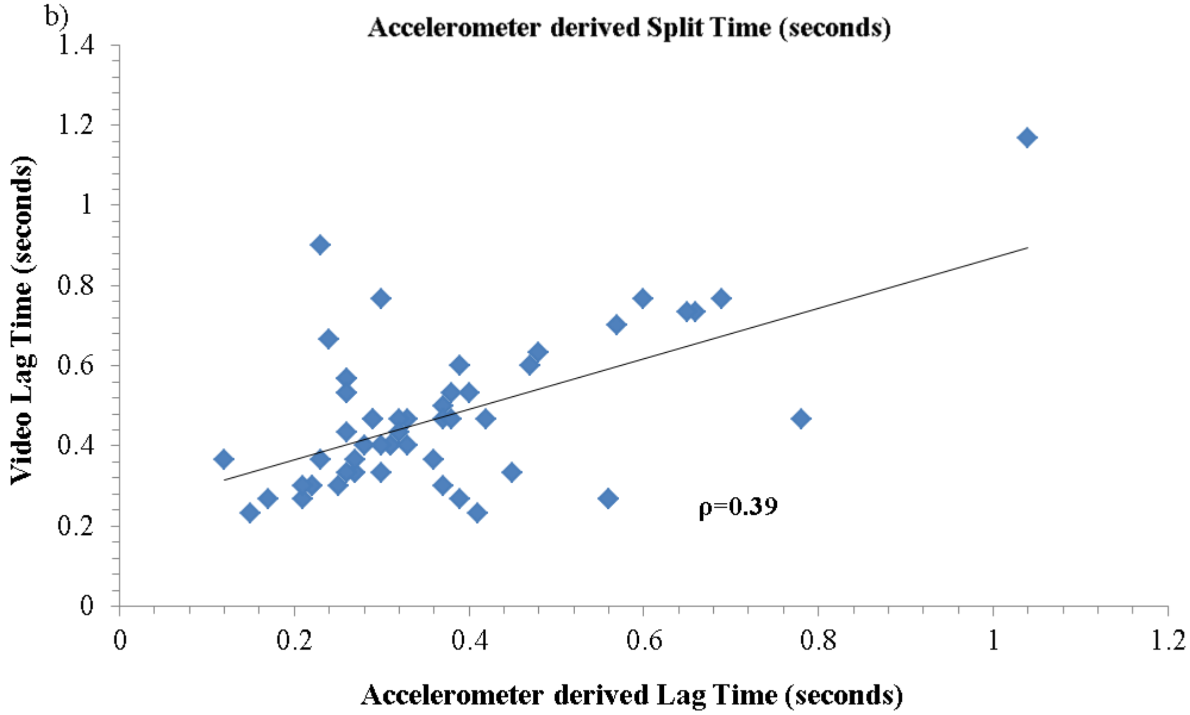

Supplementary Figure 4a, 4b. Spearman rho correlations for Split Time and Lag Time (seconds) measured using Accelerometer derived event criterion and compared to Video based times. A moderate correlation ($\rho=0.60$, $p<0.01$) for Split Time was observed when comparing the Accelerometer based times to the Video and a fair correlation ($\rho=0.39$, $p<0.01$) was observed when comparing Lag Time between the two measurement modalities.
